# Facile Synthesis of Gd-Functionalized Gold Nanoclusters as Potential MRI/CT Contrast Agents

**DOI:** 10.3390/nano6040065

**Published:** 2016-04-09

**Authors:** Wenjun Le, Shaobin Cui, Xin Chen, Huanhuan Zhu, Bingdi Chen, Zheng Cui

**Affiliations:** 1 The Institute for Translational Nanomedicine, Shanghai East Hospital, The Institute for Biomedical Engineering & Nano Science, Tongji University School of Medicine, Shanghai 200120, China; lwj2120@126.com (W.L.); cbin1991@hotmail.com (S.C.); xinchen513@yahoo.com (X.C.); zhuhuanhuan132@163.com (H.Z.); 2 State Key Lab of Silicon Materials, Zhejiang University, Hangzhou 310027, China; 3 Department of Pathology, Wake Forest University School of Medicine, Winston-Salem, NC 28780, USA

**Keywords:** gold nanocluster, bovine serum albumin, contrast agent, magnetic resonance imaging, computed tomography

## Abstract

Multi-modal imaging plays a key role in the earlier detection of disease. In this work, a facile bioinspired method was developed to synthesize Gd-functionalized gold nanoclusters (Gd-Au NCs). The Gd-Au NCs exhibit a uniform size, with an average size of 5.6 nm in dynamic light scattering (DLS), which is a bit bigger than gold clusters (3.74 nm, DLS), while the fluorescent properties of Gd-Au NCs are almost the same as that of Au NCs. Moreover, the Gd-Au NCs exhibit a high longitudinal relaxivity value (r1) of 22.111 s^−1^ per mM of Gd in phosphate-buffered saline (PBS), which is six times higher than that of commercial Magnevist (A complex of gadolinium with a chelating agent, diethylenetriamine penta-acetic acid, Gd-DTPA, r1 = 3.56 mM^−1^·s^−1^). Besides, as evaluated by nano single photon emission computed tomography (SPECT) and computed tomography (CT) the Gd-Au NCs have a potential application as CT contrast agents because of the Au element. Finally, the Gd-Au NCs show little cytotoxicity, even when the Au concentration is up to 250 μM. Thus, the Gd-Au NCs can act as multi-modal imaging contrast agents.

## 1. Introduction

Multi-modality imaging is now commonplace in clinical practice [1], especially in the field of nuclear medicine, positron emission tomography/computed tomography (PET/CT) and single-photon emission computed tomography (SPECT)/CT [2]. The current method relies on positron emission tomography (PET), which is expensive and exposes people to radiation, and these are undesirable features for a population screening method [3]. However, the conundrum of modality selection in clinical diagnostic imaging is that modalities with the highest sensitivity have relatively poor resolution, while those with high resolution have relatively poor sensitivity [4].

Magnetic resonance imaging (MRI), as a common and cheaper imaging technology since 1970s, has become a powerful imaging modality for clinical diagnostic imaging, depending on the advantages of being non-invasive, having no ionizing radiation, and having unlimited depth of tissue penetration and high spatial resolution, especially for soft tissues [5,6,7,8]. The most extensively currently used contrast agents in the clinic are paramagnetic gadolinium (Gd) chelates, such as Gd-DTPA (Magnevist^®^, Schering AG, Berlin, Germany). Gd chelates can change the signal intensity by shortening the longitudinal relaxation time (T1) of water molecules [9]. Unfortunately, due to fast renal clearance resulting from the low molecular weight, the biomedical applications of Gd chelates are discouraged by the short intrinsic response time and non-specificity to target organs [10]. Therefore, designing and developing an alternative approach with non-toxicity, prolonged residence time, and specific distribution has become critically important [11,12,13].

Computed tomography (CT) has the advantages of rapid image acquisition and high contrast and spatial resolution; the advent of it has revolutionized diagnostic radiology [14,15]. However, it also has some limitations. For instance, it is an increasing source of radiation exposure to patients. In addition, it has a superb three-dimensional (3D) resolution for structure imaging but is short on tissue sensitivity for functional imaging [16]. Multi-modality imaging with two or more imaging modalities can therefore allow the integration of strengths of individual modalities while overcoming their limitations [17,18]. CT and MRI, for example, provide unparalleled structural detail in anatomical imaging technologies [19].

Recently, the development of new multi-functional bionanocomposites shows a promising research topic due to the properties inherent from the biopolymers with biocompatibility and biodegradability [20]. Bovine serum albumin (BSA), as a common and economical available biopolymer, has been widely applied in the preparation of bionanocomposites (e.g., Au nanoclusters [21], CdSe [22], Cu nanoclusters [23,24], etc.) for in vivo bioapplications during the past few years. The preparations of the above nanoclusters have a lot of advantages, such as milder reaction conditions, facile processing, good reproducibility, biocompatibility, and robust stability. Inspired by the above-mentioned reports, in this study, we report a facile, one-pot route for the preparation of Gd-Au nanoclusters (Gd-Au NCs) through an albumin-mediated strategy. The obtained Gd-Au NCs exhibited a pronounced elevation of higher longitudinal relaxivity (r1 = 22.111 mM^−1^·s^−1^) than Magnevist (r1 = 3.56 mM^−1^·s^−1^). Consequently, it can potentially be employed for multi-modal imaging contrast agents.

## 2. Results and Discussion

### 2.1. Preparation of Gd-Au NCs

In bioinspired biomineralization, the biomacromolecules are used to collect and transport raw materials and assemble them into ordered composites with consistency and uniformity in an aqueous environment under mild conditions [13]. BSA as a biomacromolecule is commonly employed as a template in biomineralization. The molecular structure of BSA includes lots of disulfide bonds (among the 17 available per molecular), and these bonds have a strong affinity with the surface of metal atoms [25]. At the same time, some amino acids (e.g., tyrosine) of BSA possess strong reducibility under alkaline conditions. In the experiment, Au NCs were synthesized according to an improved “green” synthetic route [21]. The pH values of the solutions were adjusted to 12 to trigger the reduction capability of the responsible amino acids, and then the solutions were maintained at 37 °C for 12 h to ensure the complete reduction of HAuCl_4_.

In the synthesis of Gd-Au NCs, aqueous gadolinium chloride solution is introduced to the above reaction system. Briefly, aqueous gadolinium chloride solution was mixed with HAuCl_4_ solution under vigorous stirring. Then, BSA solution was added to the mixture under the same condition. Ten minutes later, NaOH solution was utilized to adjust the pH values of the reaction solution, and the mixture was subsequently stirred at 37 °C (water bath). The color of the solution would change from light yellow to deep brown, which indicated that the Gd-Au nanoclusters were formed and they tended to be stable. Purification of Gd-Au NCs was firstly performed by dialysis to remove the small molecules, included some metal ions. The residue was subsequently freeze-dried from liquid to solid. Finally, the powder was dispersed in phosphate-buffered saline (PBS, 0.01 M, pH 7.4) and stored at 4 °C for further study.

### 2.2. Characterization of Gd-Au NCs

**Figure 1:**
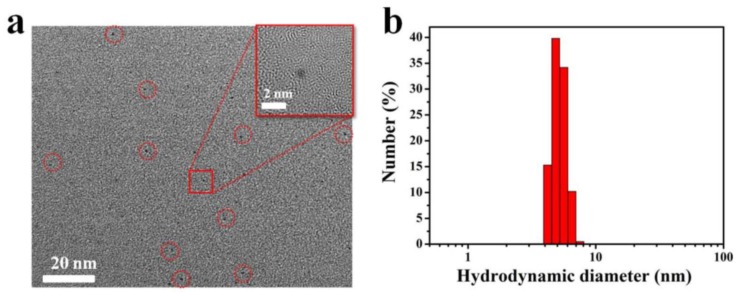
(**a**) Transmission electron microscopy image of as-prepared Gd-Au nanoclusters; (**b**) The result of dynamic light scattering.

**Figure 2:**
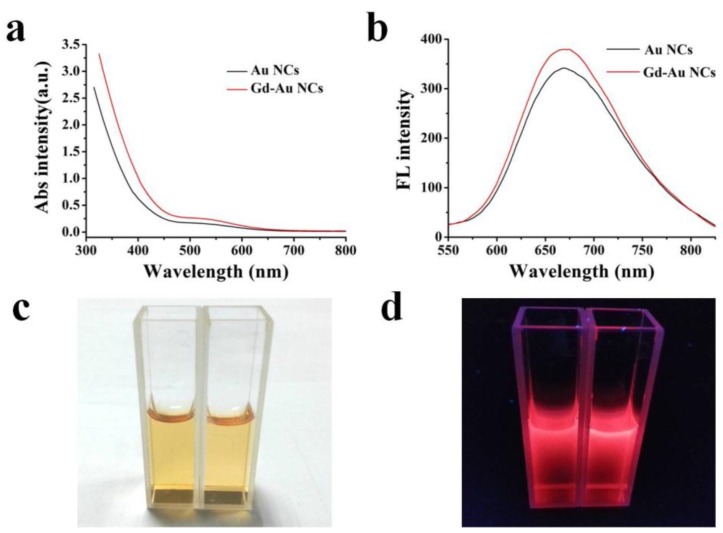
(**a**) Ultraviolet–visible absorption spectra of Au nanoclusters (NCs) (black curve) and Gd-Au NCs (red curve), Abs (Absorbance), a.u. (Absorbance Unit); (**b**) The corresponding fluorescent emission spectra of Au NCs (black curve) and Gd-Au NCs (red curve); (**c**) Bright photograph of Au NCs (left) and Gd-Au NCs (right); (**d**) The corresponding fluorescent photograph of Au NCs (left) and Gd-Au NCs (right) was taken under a porTable 365 nm UV-lamp (Min hang, Shanghai, China).

The Gd-Au NCs were synthesized in one step, and characterized by transmission electron microscopy (TEM), dynamic light scattering (DLS), fluorescent emission spectra, and ultraviolet–visible (UV-vis) spectroscopy, *etc.* As shown in Figure 1, the TEM image (Figure 1a) and the results of DLS (Figure 1b) consistently disclose that Gd-Au NCs exhibit a uniform size, with an average hydrodynamic diameter of 5.6 nm, which is a bit bigger than that of gold clusters (3.74 nm, DLS) reported in the literature [26]. The size of the nanoclusters increased, and this is likely due to the result of the Gd ion being directly involved in the formation of clusters. Therefore, in this study, we subsequently investigated the influence of gadolinium salt on the fluorescent properties of Au NCs (Figure 2). UV-vis absorption and the corresponding fluorescent emission spectra of Au NCs and Gd-Au NCs are shown in Figure 2a,b. The as-prepared nanoclusters have a photoemission peak at ~670 nm. The bright photograph and the corresponding fluorescent photograph of Au NCs and Gd-Au NCs are shown in Figure 2c,d. No obvious change in fluorescence properties was observed between Au NCs and Gd-Au NCs in solutions under the same concentrations of Au from the inductively coupled plasma mass spectrometry (ICP-MS) measurement. In addition, we found that the molar ratio of Au:Gd elements in Gd-Au NCs solutions was 1:1.09.

The oxidation states of the Gd-Au NCs and Au NCs were determined by X-ray photoelectron spectroscopy (XPS). The XPS measurement was performed on a Perkin-Elmer PHI5300 spectrometer (Waltham, MA, USA). Figure S1 demonstrates the photoelectron spectra of the Gd-Au NCs and Au NCs. The 4f_7/2_ and 4f_5/2_ binding energy values of gold appeared at 83.2 eV and 87.1 eV (Figure S1a), respectively. In addition to the gadolinium peak of the Gd 4d region (Figure S1b), the region also matched well with the data reported on Gd-doped CeO_2_ [27]. If they were only chelated to BSA on the Au surface, it would involved a spin-orbit coupled 3_d5/2_ and 3d_3/2_ doublet with a binding energy position of 1186 eV and 1218 eV [28], respectively. Meanwhile, the XPS of Au NCs are shown in Figure S1c,d. The 4f_7/2_ and 4f_5/2_ binding energy values of gold appeared at 84.2 eV and 87.6 eV (Figure S1a), respectively. In addition, there was no characteristic peak for the 4d region of Gd in XPS of Au NCs. The above results may indicate that the gadolinium was successfully involved in the formation of Au clusters.

### 2.3. MRI/CT in Vitro

**Figure 3:**
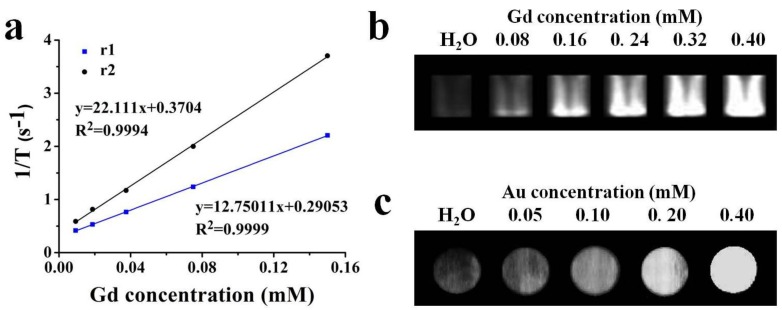
(**a**) Longitudinal (T1) and transverse (T2) relaxation times of Gd-Au NCs (the slopes response to the longitudinal relaxivity value r1( blue) and transverse relaxivity value r2 (black)); (**b**) Magnetic resonance images of the Gd-Au NCs with Gd concentrations ranging from 0.08 to 0.40 mM and H_2_O; (**c**) Computed tomography of the Gd-Au NCs containing various Au concentrations and H_2_O.

To evaluate the capacity of Gd-Au NCs as an effective the longitudinal relaxation time (T1)-weighted MRI contrast agent, the longitudinal (T1) and transverse (T2) relaxation times were measured with a 1.5 T NMR analyzer (Milton, ON, Canada) at different Gd concentrations (9.325, 18.75, 37.5, 75, 150 μM) from the ICP-MS measurement, respectively. As shown in Figure 3a, the Gd-Au NCs exhibited a high r1 value of 22.111 s^−1^ per mM of Gd in PBS, which is six times higher than that of commercial Magnevist (Gd-DTPA, r1 = 3.56 mM^−1^·s^−1^) [12]. The significant increasing may be due to the favorable water solubility, the small size, and the confined tumbling in the biomacromolecule, resulting in a longer rotational correlation time [29,30]. In addition to the improved longitudinal relaxivity (r1), the relatively low ratio (r2/r1 = 1.73 < 3) is beneficial in producing a desired T1 positive contrast effect [31]. To explore the potential of Gd-AuNCs as MRI contrast agents, different Gd concentrations (0, 0.08, 0.16, 0.24, 0.32, 0.40 mM) were evaluated by a 3.0 T clinical MR scanner (GE, Milwaukee, WI, USA) at 25 °C. T1-weighted magnetic resonance (MR) images and the corresponding signal intensity in Figure 3b further confirmed that the Gd-Au NCs exhibited an enhanced T1 signal, demonstrating that they can act as a highly efficient T1-enhanced MR contrast agent *in vitro*.

The Au element has higher X-ray attenuation than iodine due to its higher atomic number and electron density [19]. Furthermore, to confirm the feasibility of Gd-Au NCs for CT contrast agents, different Au concentrations (0, 0.05, 0.1, 0.20, 0.40 mM) from the ICP-MS measurements were evaluated by Nano SPECT/CT (Washington, DC, USA). As shown in Figure 3c, the as-prepared Gd-Au NCs have a higher signal than H_2_O, and as the concentration of gold increased, its corresponding CT signal gradually improved, which reveals that the Gd-Au NCs can also be a potential CT contrast agent *in vitro*.

### 2.4. In Vitro Cytotoxicity

**Figure 4:**
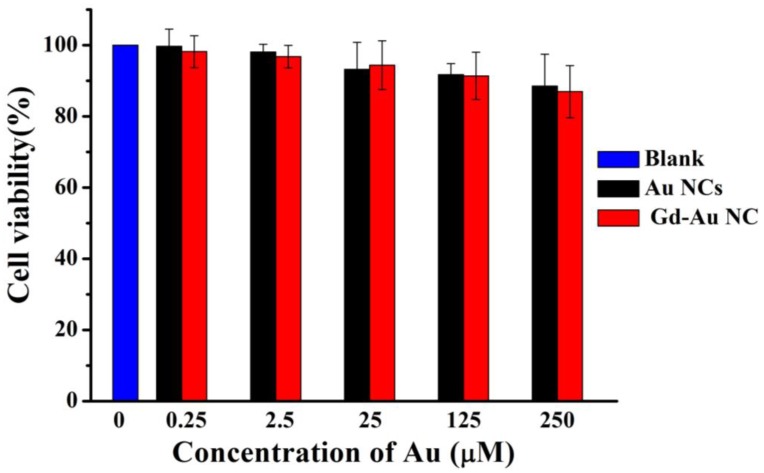
*In vitro* cytotoxicity of Au NCs (black) and Gd-Au NCs (red) against breast cancer cell line (MCF-7) after 24 h.

The cytotoxicity of Gd-Au NCs was evaluated via cell counting kit-8 (CCK-8) assay by incubating the breast cancer cell line (MCF-7) with Au NCs and Gd-Au NCs at various concentrations of Au for 24 h, respectively. The CCK-8 results in Figure 4 indicated that the Gd-Au NCs showed little cytotoxicity against MCF-7 cells, even at Au concentrations up to 250 μM. Also, there is no significant difference in cell viability between Au NCs and Gd-Au NCs. This at least indicated that the gadolinium ions have a strong bond with BSA molecules and will not obviously increase the cytotoxicity of nanoclusters in the cell culture system. These results showed that Gd-Au NCs were of low toxicity and safe against the MCF-7 cells at the test concentrations. This was in accordance with the non-toxicity, low immunogenicity, and good biocompatibility and biodegradability of both Au NCs and Gd-BSA as previously reported in literature [13,28].

## 3. Materials and Methods

### 3.1. Materials

All initial reagents were obtained commercially and used as received. Albumin from bovine serum (BSA) was purchased from Sigma-Aldrich (Louis, MO, USA). The HAuCl_4_ were purchased from Guoyao Reagent Corporation (Shanghai, China). MCF-7 cell line was purchased from Shanghai Institute of Cell Biology (Shanghai, China). Dulbecco’s modified eagle’s medium (DMEM), Fetal bovine serum (FBS), Phosphate-buffered saline (PBS) and 0.25% Trypsin-EDTA were purchased from Gibco Corp (Grand Island, NY, USA). Millipore ultrapure water (Billerica, MA, USA) (18.2 MΩ·cm resistivity at 25 °C) was used throughout the entire experiments.

### 3.2. Preparation of Gd-Au NCs

In a typical experiment, aqueous gadolinium chloride solution (0.15 mL, 500 mM) was added to HAuCl_4_ solution (5 mL, 10 mM) slowly under vigorous stirring. Then BSA solution (5 mL, 50 mg/mL) was added to the mixture under vigorous. Ten minutes later, NaOH solution (0.75 mL, 1 M) was introduced under ultrasonic dispersion and the mixture was continuously stirred at 37 °C for 12 h under nitrogen. During this period, the color of the solution changed from light yellow to light brown, and then to deep brown. Purification of Gd-Au NCs was performed by dialysis to remove the small molecules and was freeze-dried from liquid to solid. Finally, the powder was dispersed in PBS (0.01 M, pH 7.4) and stored at 4 °C for further study.

BSA-stabilized Au clusters (Au NCs) were prepared according to an improved ‘green’ synthetic route [21].

### 3.3. Characterization of Gd-AuNCs

Transmission electron microscopy (TEM) images of Gd-Au NCs were obtained using a JEM-2100F electron microscope (JEOL Ltd., Tokyo, Japan) working at 200 kV. The nanoparticles were dispersed in deionized water (DIW) and dried onto carbon-coated copper grids. Then the air-dried samples were directly observed by electron microscope. The hydrodynamic diameter analyses of the aqueous were performed on a laser light scattering system (JEM Zetasizer Nano-ZS90, Great Malvern, England, UK). UV-vis absorption and fluorescent emission spectra were measured by Cary 50 spectrophotometer (Varian, Palo Alto, CA, USA) and F-182 4500 spectrophotometer (Hitachi, Chiyoda, Tokyo, Japan), respectively. The concentration of Gd/Au was measured with ICP-AES (P-4010, Hitachi, Chiyoda, Tokyo, Japan).

### 3.4. Relaxometry and MRI in Vitro

The longitudinal (T1) and transverse (T2) relaxation times of Gd-Au NCs were measured with a 1.41 T minispec mq 60 NMR Analyzer (Bruker, Germany) at 37 °C. The MR phantom images *in vitro* were acquired using a 3.0 T Sigma scanner (GE, Milwaukee, WI, USA). The T1-weighted MR images of Gd-Au NCs were obtained with different Gd concentrations (0, 0.08, 0.16, 0.24, 0.32, 0.40 mM) using Tl-weighted pulse sequences, respectively. The measurement parameters were as follows: T1-weighted sequence, spin echo (SE), the repetition time (TR) and the echo time (TE) = 500/18.2 ms), matrix acquisition = 90 × 90, number of complex samples (NS) = 2, field-of-view (FOV) = 80 mm × 80 mm, slices = 1, slice width = 5.0 mm, slice gap = 0.55 mm, 0.55 T, 32.0 °C. Relaxivity values of r1 and r2 were calculated by fitting the 1/T1 and 1/T2 relaxation time (s^−1^) *versus* Gd concentration (mM) curves.

### 3.5. In Vitro Cytotoxicity

MCF-7 cell line was cultured in a 37 °C incubator with 5% CO_2_ in Dulbecco’s Modified Eagle Medium (DMEM) supplemented with 10% FBS, streptomycin at 100 mg/mL and penicillin at 100 U/mL. The *in vitro* cytotoxicity of Gd-Au NCs was measured using a standard cell counting kit-8 (CCK-8) assay. Typically, MCF-7 cells (5 × 10^3^/well) were seeded into a 96-well plate (three parallel holes per group), and incubated in the culture medium for 12 h at 37 °C under 5% CO_2_. The culture medium was then removed, and cells were incubated with fresh culture medium containing 100 μL of Au NCs and Gd-Au NCs at varied Au concentrations (0.25 μM, 2.5 μM, 25 μM, 125 μm, 250 μM) at 37 °C under 5% CO_2_ for additional 24 h, respectively. Then 10 μL of CCK-8 agentia (5 mg/mL) was added into the plates and incubating cells for further 3 h. In the end, the OD450 value (Absolute values) of each well was measured using the multifunction microplate reader (Tecan infinite M200 Pro, Tecan Group Ltd., Männedorf, Switzerland).

## 4. Conclusions

In conclusion, we synthesized Gd-Au NCs using bioinspired biomineralization. The particle size of Gd-Au NCs is a bit bigger than that of Au NCs because of the Gd involvement in the formation of clusters, while the Gd-Au NCs exhibit excellent fluorescent properties that are almost the same as that of Au NCs. The *in vitro* MRI results show that the r1 value of the Gd-Au NCs is six times higher than that of commercial Magnevist (Gd-DTPA), which is mainly due to the favorable water solubility, the small size, and the confined tumbling in a biomacromolecule. Furthermore, the Gd-Au NCs can be used as a CT contrast agent because of the Au element, as evaluated by Nano SPECT/CT. Moreover, the Gd-Au NCs show non-toxicity and good biocompatibility. All these results indicate that Gd-Au NCs are promising for use as a multi-modal imaging contrast agent.

## Supplementary Materials

The following are available online at http://www.mdpi.com/2079-4991/6/4/65/s1.
