# Supplementary material for: Facile Synthesis of Gd-Functionalized Gold Nanoclusters as Potential MRI/CT Contrast Agents
Source: Nanomaterials (Basel). 2016 Apr 9;6(4):65. doi: 10.3390/nano6040065 (PMC5302577; doi:10.3390/nano6040065)
Supplement: Supplementary file 1 [file nanomaterials-06-00065-s001.pdf]

Supplementary Materials: Facile Synthesis of Gd-Functionalized Gold Nanoclusters as Potential MRI/CT Contrast Agents

Wenjun Le, Shaobin Cui, Xin Chen, Huanhuan Zhu, Bingdi Chen and Zheng Cui

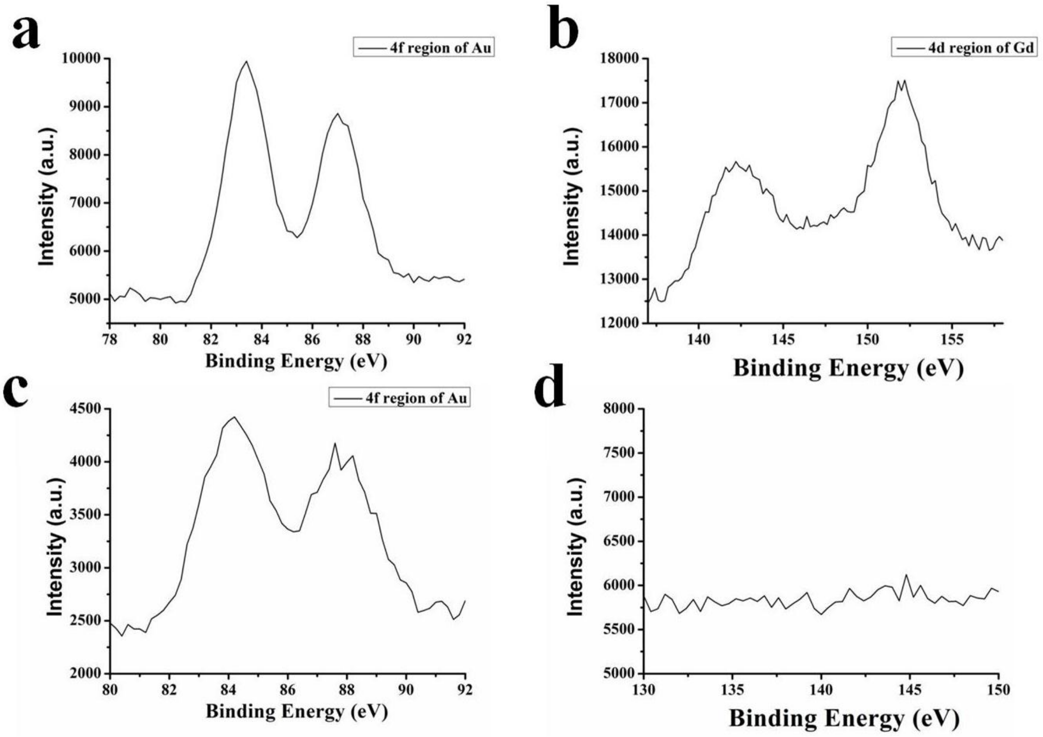

Figure S1. (**a**) X-ray photoelectron spectroscopy (XPS) spectra of Au 4f for Gd-Au nanoclusters (NCs); (**b**) the 4d region of Gd for Gd-Au NCs; (**c**) XPS spectra of Au 4f for Au NCs; (**d**) the blinding energy region of Au NCs. (a.u.: Absorbance Unit).

© 2016 by the authors; licensee MDPI, Basel, Switzerland. This article is an open access article distributed under the terms and conditions of the Creative Commons by Attribution (CC-BY) license (http://creativecommons.org/licenses/by/4.0/).
